# Impact of α-synuclein fibril structure on seeding activity in experimental models of Parkinson’s disease

**DOI:** 10.1038/s41531-025-01080-2

**Published:** 2025-07-31

**Authors:** Junichiro Ohira, Masanori Sawamura, Kenichi Kawano, Risa Sato, Tomoyuki Taguchi, Tomoyuki Ishimoto, Jun Ueda, Masashi Ikuno, Shu-ichi Matsuzawa, Katsumi Matsuzaki, Ryosuke Takahashi, Hodaka Yamakado

**Affiliations:** 1 Department of Neurology, Kyoto University Graduate School of Medicine, Kyoto, Japan; 2 Graduate School of Pharmaceutical Sciences, Kyoto University, Kyoto, Japan; 3 Department of Therapeutics for Multiple System Atrophy, Kyoto University Graduate School of Medicine, Kyoto, Japan; 4 Kyoto University Office of Research Acceleration (KURA), Kyoto, Japan

**Keywords:** Molecular biology, Neurological disorders

## Abstract

The central pathogenesis of Parkinson’s disease involves the misfolding and aggregation of α-synuclein (α-syn). There is a widespread belief that α-syn can propagate in a prion-like manner, and α-syn preformed fibrils (PFFs) have been widely used to establish α-syn propagation models. However, achieving standardized protocols for generating PFFs is challenging due to the influence of various factors on propagation efficiency, resulting in inter-laboratory and inter-experimental variability. Among these factors, the size of the PFFs is considered the most influential as unsonicated PFFs exhibit limited seeding and propagation abilities. Therefore, the objective of our research is to examine the impact of the size and conformation of sonicated PFFs on seeding activity. PFFs were sonicated under various conditions using a conventional water bath sonicator and a high-power sonicator, which is commonly used for DNA shearing in next-generation sequencing. Each sonicated PFF was analyzed for in vitro/in vivo seeding activities, after size confirmation by electron microscopy and a conformational analysis by Fourier Transform Infrared (FTIR) spectroscopy. Strong sonication for 30 min generated extremely short fibrils with the highest seeding activity, which is the optimal condition for the propagation model, whereas sonication for 60 minutes or more led to a reduction in seeding activity. FTIR spectroscopy suggested that sonication disrupted the aggregated strands and generated new fibril ends, thereby accounting for the increased seeding activity; however, prolonged sonication for 60 min or more released monomers with disrupted β-sheet structure from PFFs and reduced the seeding activity. In conclusion, the balance between size reduction and preservation of the β-sheet structure in PFFs plays a critical role in seeding activity. Optimizing these parameters of α-syn PFFs can help improve reproducible preclinical animal models based on α-syn propagation.

## Introduction

Parkinson’s disease (PD) is a neurodegenerative disorder characterized by the progressive loss of dopaminergic neurons and the formation of intracellular inclusions called Lewy bodies (LBs) composed primarily of aggregated α-synuclein (α-syn)^1,2^. Recent studies have shown that α-syn has prion-like properties and can propagate between neurons in vivo, which may explain the continuous progression of Lewy pathologies in the Braak hypothesis^3–11^. Generating an in vivo model based on the α-syn propagation hypothesis is expected to help recapitulate the entire natural history of PD and facilitate the development of disease-modifying therapies based on the PD pathogenesis.

α-syn preformed fibrils (PFFs) generated from recombinant monomers have been widely used to create α-syn propagation models^12–16^. However, the generation of α-syn PFFs and their administration methods are not standardized, often resulting in inter-laboratory and inter-experimental variability due to differences in propagation efficiency^6,17–19^. Many factors affect propagation efficiency, such as the species^20^ and buffer conditions that produce α-syn PFFs^18^, but the most influential one is the PFF size, since non-sonicated α-syn fibrils are unlikely to propagate^17,21^. Sonicated α-syn PFFs can be internalized by neurons and serve as a template for subsequent fibrillation. PFFs are known to elongate with the addition of monomers to their ends, and sonication has been shown to increase fibril ends, thereby contributing to increased seeding activity^22–24^. Establishing an improved and reproducible α-syn propagation model will require careful examination of the factors that influence the propagation efficacy.

In the present study, we investigated the effect of sonication on the seeding activity of α-syn mouse PFFs (mPFFs) and, in part, human PFFs (hPFFs) using propagation models with a high-power sonicator commonly used for DNA shearing in next-generation sequencing and compared it with a conventional water bath sonicator. Analysis of sonicated PFFs by electron microscopy and Fourier-transform infrared (FTIR) spectroscopy, as well as monomer release experiment from PFFs, revealed that the balance between the size and preservation of β-sheets in PFFs is critical for in vivo seeding activity and in vitro propagation efficiency.

## Results

### The high-power sonicator produces more fragmented fibrils than the conventional sonicator

**Fig. 1:**
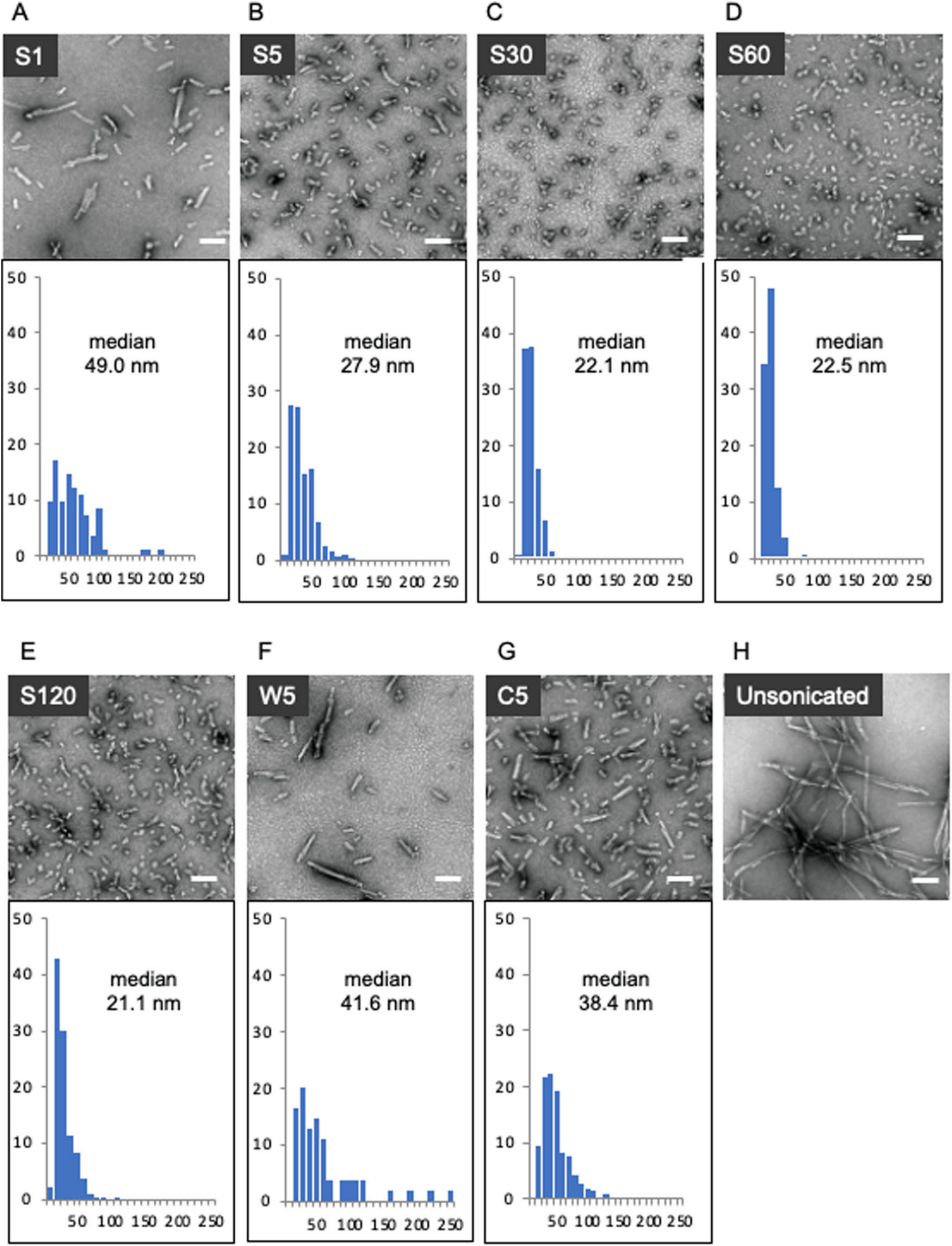
Transmission electron microscopy of sonicated α-syn mPFFs reveals that the high-power sonicator produces much shorter PFFs than the conventional sonicator. **A**–**F** α-syn mPFFs after 1 min (S1), 5 min (S5), 30 min (S30), 60 min (S60), or 120 min (S120) of sonication under strong conditions of a high-power sonicator, or after 5 min of sonication (W5) under weak conditions. **G** mPFFs after 5 min of sonication in a conventional bath sonicator (C5). **H** Unsonicated mPFFs. **A**–**G** Histograms and median PFF lengths are shown below the electron microscope images. Scale bars: 100 nm.

mPFFs sonicated under various conditions, and those without sonication were examined with an electron microscopy, and their lengths were measured (Fig. 1A–H). When the same 5 min sonication was performed under the strong condition of the high-power sonicator (S5, Fig. 1B), weak condition of the high-power sonicator (W5, Fig. 1F), and conventional bath sonicator (C5, Fig. 1G), the mean length of S5 was significantly shorter than W5 and C5 (*p* < 0.001). Next, the mPFF lengths were compared by varying the sonication time under the strong condition of the high-power sonicator. The length decreased in a time-dependent manner for up to 30 min of sonication (Figs. 1C, S30), but no further decrease was observed, even when the sonication time was increased to 60 or 120 min (Fig. 1D, E). Sonication with the high-power sonicator for 30 min or more produced mPFFs with a median length of approximately 22 nm, which is much shorter than that previously reported^12,14,25^. Experiments using hPFFs were conducted in the same manner as with mPFFs; with two modifications: immunocytochemistry (ICC) of the primary cultures was performed one week after hPFF administration, and C30 was used instead of W5 (Fig. S1). The fibril length decreased in a time-dependent manner up to 120 min under strong sonication (Fig. S1A–E). When comparing 5 min and 30 min sonication between the high-power sonicator (S5, S30) and the conventional bath sonicator (C5, C30), the mean lengths of S5 and S30 were significantly shorter than those of C5 and C30 (*p* < 0.001).

### More fragmented PFFs produced stronger phosphorylated α-syn pathology in vitro

**Fig. 2:**
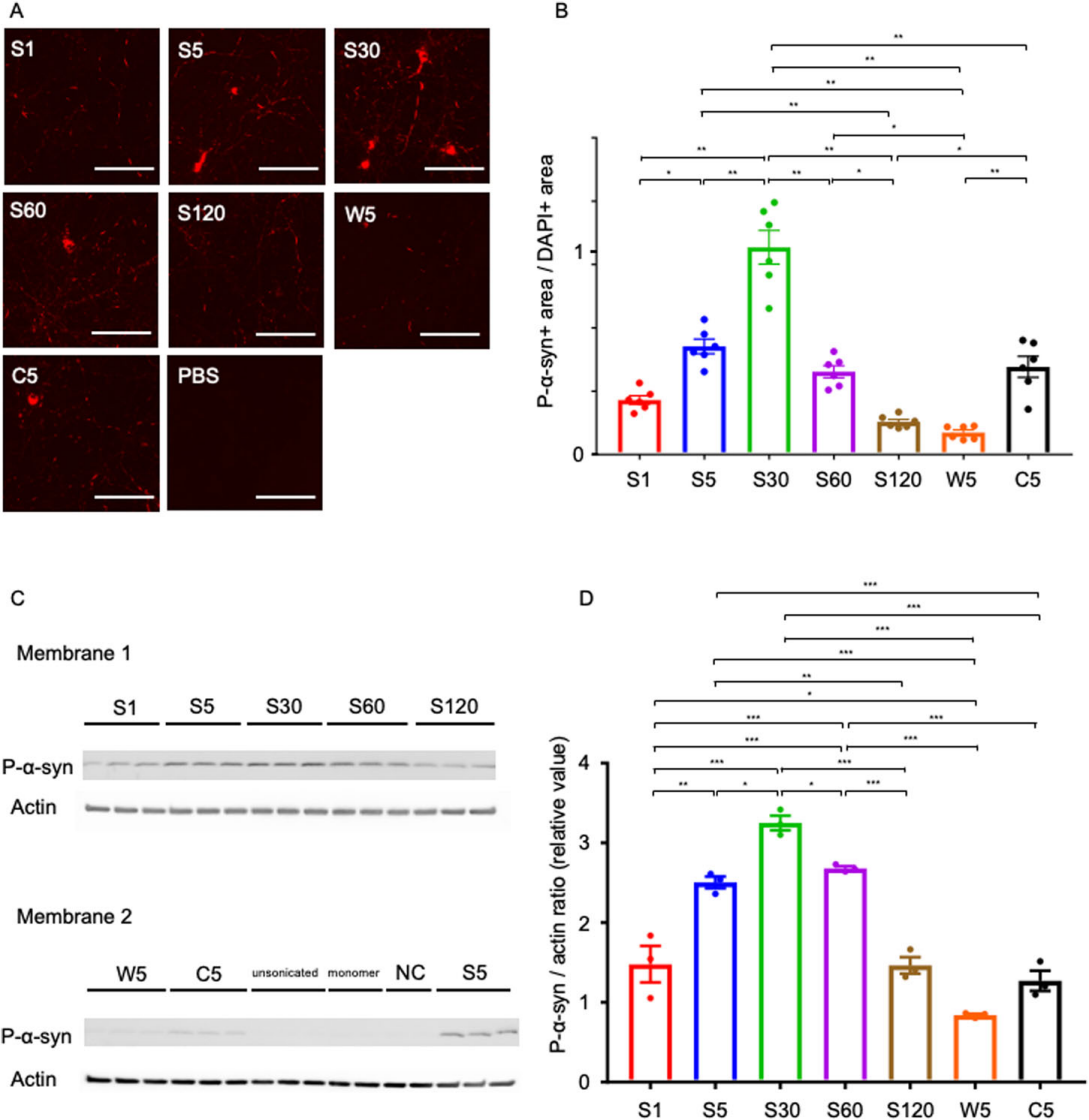
Shorter α-syn mPFFs show a higher seeding activity in primary neurons. **A** Representative images of α-syn aggregates in mouse primary hippocampal neurons 3 days after the addition of each mPFF, using an antibody against p-α-syn. Scale bars: 100 nm. **B** P-α-syn positive area divided by the DAPI-positive area to normalize for the number of cells. The average value for S30 was set to 1 on the vertical axis. S30 forms the strongest pathology. Each plotted dot represents the average from four independent well regions, and six independent experiments were analyzed. A one-way ANOVA with Tukey’s multiple-comparisons test is performed; *p < 0.05, **p < 0.0001. Data are expressed as the mean ± SEM. **C** Western blot analysis using a p-α-syn-specific antibody. p-α-syn pathology worsened in a time-dependent manner up to 30 min of sonication but decreased after 60 and 120 min of sonication (*n* = 3 for sonicated or unsonicated mPFFs, and *n* = 2 for monomer or negative control). **D** Densitometric analysis of (**C**). The total density of each sample is measured and normalized against actin levels. A one-way ANOVA with Tukey’s multiple-comparisons test is performed; *p < 0.05, **p < 0.005, ***p < 0.0001.

The majority of α-syn found in Lewy pathology is phosphorylated at Ser129, and thus p-α-syn can serve as a useful pathological marker for PD models^8,10,11,26^. Primary neurons transduced with mPFFs (final concentration: 0.5 µg/mL) form aggregates positive for p-α-syn and have been established as an in vitro model for prion-like propagation of α-syn in PD. Each sonicated mPFF was added to mouse primary hippocampal neurons (*n* = 6), and the ability to form aggregates was analyzed by immunocytochemistry using a p-α-syn-specific antibody after 3 days. The p-α-syn pathology worsened in a time-dependent manner up to 30 min of sonication, but decreased inversely after 60 and 120 min of sonication (Fig. 2A, B). We confirmed reproducibility through multiple experiments and conducted immunocytochemistry using the same fresh α-syn PFFs as in the monomer release experiments described later, yielding similar results (data not shown). The trend observed in hPFFs was consistent with that seen in mPFFs (Fig. S2). P-α-syn pathology progressively increased with sonication time up to 30 min, but subsequently exhibited a sharp decline at 60 and 120 min. Although p-α-syn pathology was stronger in C30 than in C5, the difference was not statistically significant.

Additionally, the ability to form aggregates was analyzed by western blot using a p-α-syn-specific antibody 3 days after each sonicated mPFF was added to mouse primary hippocampal neurons. Similar results to immunocytochemistry were obtained, indicating that p-α-syn pathology worsened in a time-dependent manner up to 30 min of sonication but decreased after 60 and 120 min of sonication (Figs. 2C, D, S3).

### More fragmented PFFs had higher seeding activity after injection into the olfactory bulb in mice

**Fig. 3:**
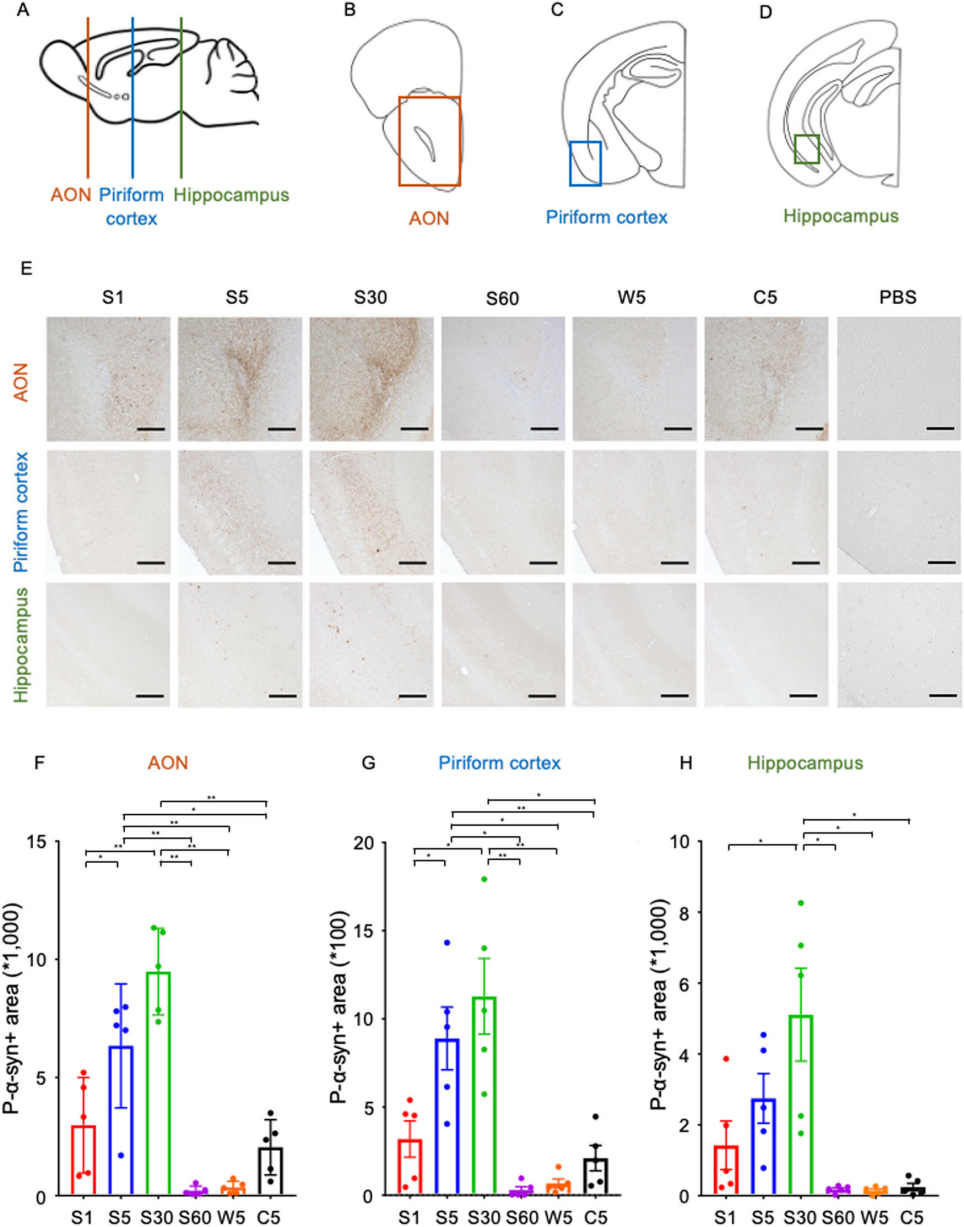
Inoculation of shorter α-syn mPFFs into the OB shows higher seeding activity in mice. **A** Sagittal sections of mouse brain. **B**–**D** Coronal section of the left brain at each level indicated in (**A**) (AON, piriform cortex, and hippocampus). Square regions are analyzed in the AON (**B**), piriform cortex (**C**), and hippocampus (**D**). **E** Representative coronal sections of the AON, piriform cortex, and hippocampus, three weeks after inoculation of each sonicated mPFF or PBS into the left OB of wild-type mice. **F**–**H** The p-α-syn-positive areas in each region are analyzed 3 weeks after inoculation of mPFFs into the left OB of mice (*n* = 5). S5 and S30 causes the most intense pathologies after inoculation into the OB of mice. A one-way ANOVA with Tukey’s multiple-comparisons test is performed; *p < 0.05, **p < 0.0001. Data are expressed as the mean ± SEM. AON, anterior olfactory nucleus. OB, olfactory bulb.

The extent of α-syn propagation was evaluated in slices of the AON, piriform cortex, and hippocampus, three weeks after each sonicated mPFF was inoculated into the left OB of wild-type mice (*n* = 6 each). These regions were selected as the primary neurons connected to the OB. Regions enclosed by squares in ipsilateral coronal sections were immunohistochemically examined using a p-α-syn antibody (Fig. 3A–D). Propagation tended to be enhanced with increasing sonication time up to 30 min (S30) in all slices, but it was drastically attenuated at S60 (Fig. 3E–H). With the same 5 min sonication, S5 exhibited significantly stronger propagation than C5 in the AON and piriform cortex and markedly stronger than W5 in all slices. Intense sonication for 30 min (S30) can create mPFFs with the strongest propagation ability, while longer sonication for more than 30 min reduces the propagation ability, in agreement with the in vitro results.

### More fragmented PFFs propagated more extensively after intranasal and gut injection in mice

**Fig. 4:**
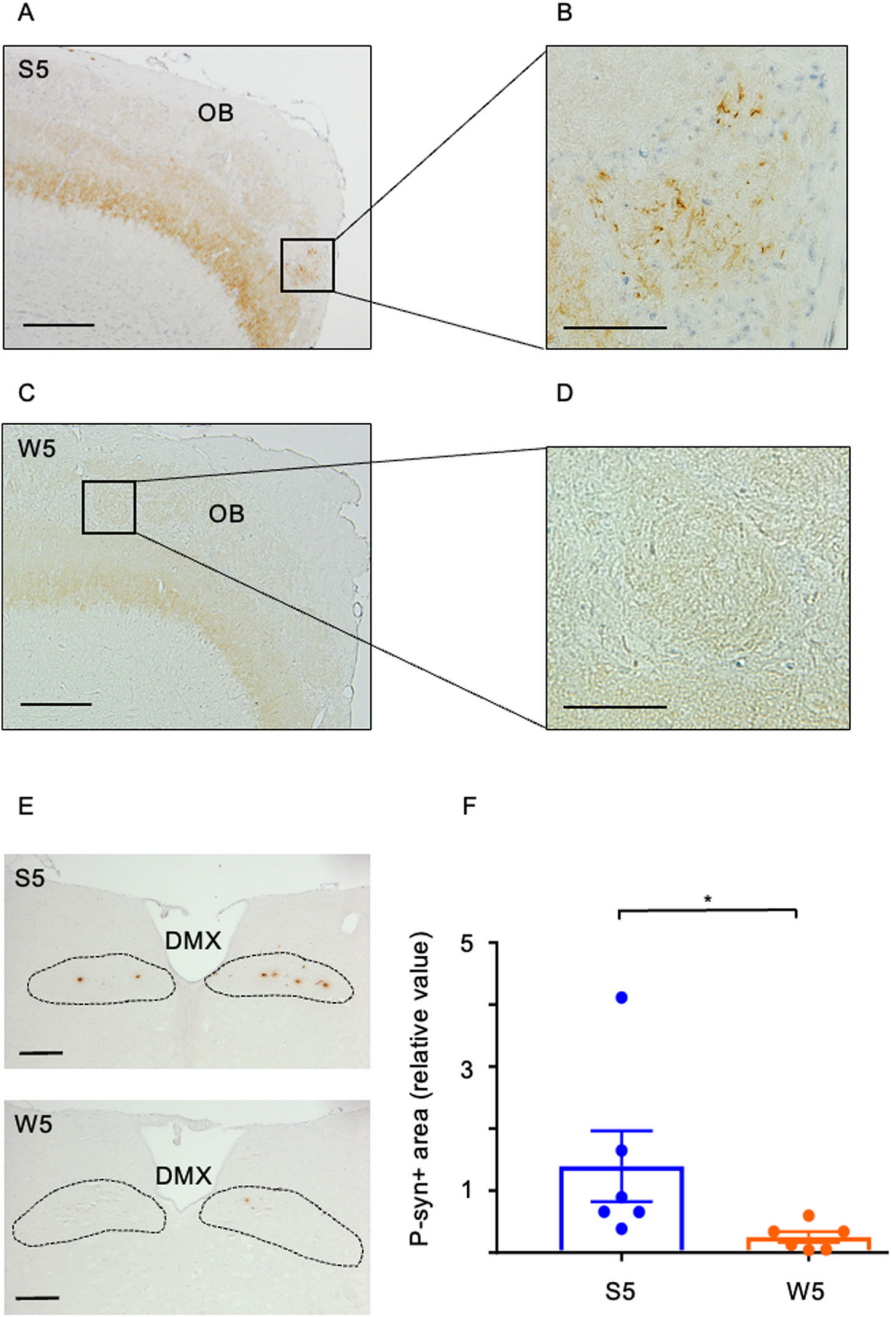
More strongly sonicated α-syn mPFFs inoculated into the nasal cavity and stomach show higher seeding activity. **A**–**D** The left OB is evaluated 2 months after intranasal administration of S5 or W5 (mPFFs) in mice (*n* = 6). **A**, **C** Representative images of the left OB showing IHC staining with antibodies against p-α-syn after S5 or W5 administration. Scale bar: 200 µm. **B**, **D** Magnified view of the glomerular layer. Scale bar: 50 µm. **E**, **F** The DMX is investigated 2 months after injection of S5 or W5 into the stomach of mice (*n* = 6). **E** Representative images showing IHC staining with antibodies against p-α-syn. Scale bar: 200 µm. **F** An analysis of the p-α-syn positive area following stomach injection of mPFFs. Propagation of S5 is stronger than that of W5. Each plotted dot represents the average of four areas, and six independent experiments are analyzed. The Mann-Whitney U test is performed; *p = 0.004.

S5 and W5 of mPFFs were administered to the left nasal cavity of six mice each, and propagation to the ipsilateral OB was assessed two months later. P-α-syn pathology was observed in the glomerular layer or olfactory sensory neurons of all six mice treated with S5 (Fig. 4A, B), whereas only one mouse treated with W5 showed p-α-syn pathology (Fig. 4C, D). No further propagation, including in the mitral cell layer, was observed. Therefore, the propagation ability of S5 was higher than that of W5 following intranasal administration.

Next, S5 and W5 of mPFFs were injected into the stomachs of six mice each, and the extent of propagation to DMX was examined two months later (Fig. 4E). The p-α-syn pathology was significantly stronger in S5 than in W5 (Fig. 4F), suggesting that S5 has a stronger propagation ability after injection into the stomach than W5. However, even S5 did not propagate to the locus coeruleus (LC) or other brain regions.

### Structural properties of sonicated PFFs that contribute to differences in seeding activity

The high-power sonicator generated shorter PFFs than the conventional sonicator, and both in vitro and in vivo experiments showed that shorter PFFs had higher seeding activity than longer PFFs. Seeding activity increased in a time-dependent manner up to 30 min of sonication under strong conditions, but decreased as sonication time increased to 60 and 120 min. The underlying mechanisms were explored in subsequent experiments.

**Fig. 5:**
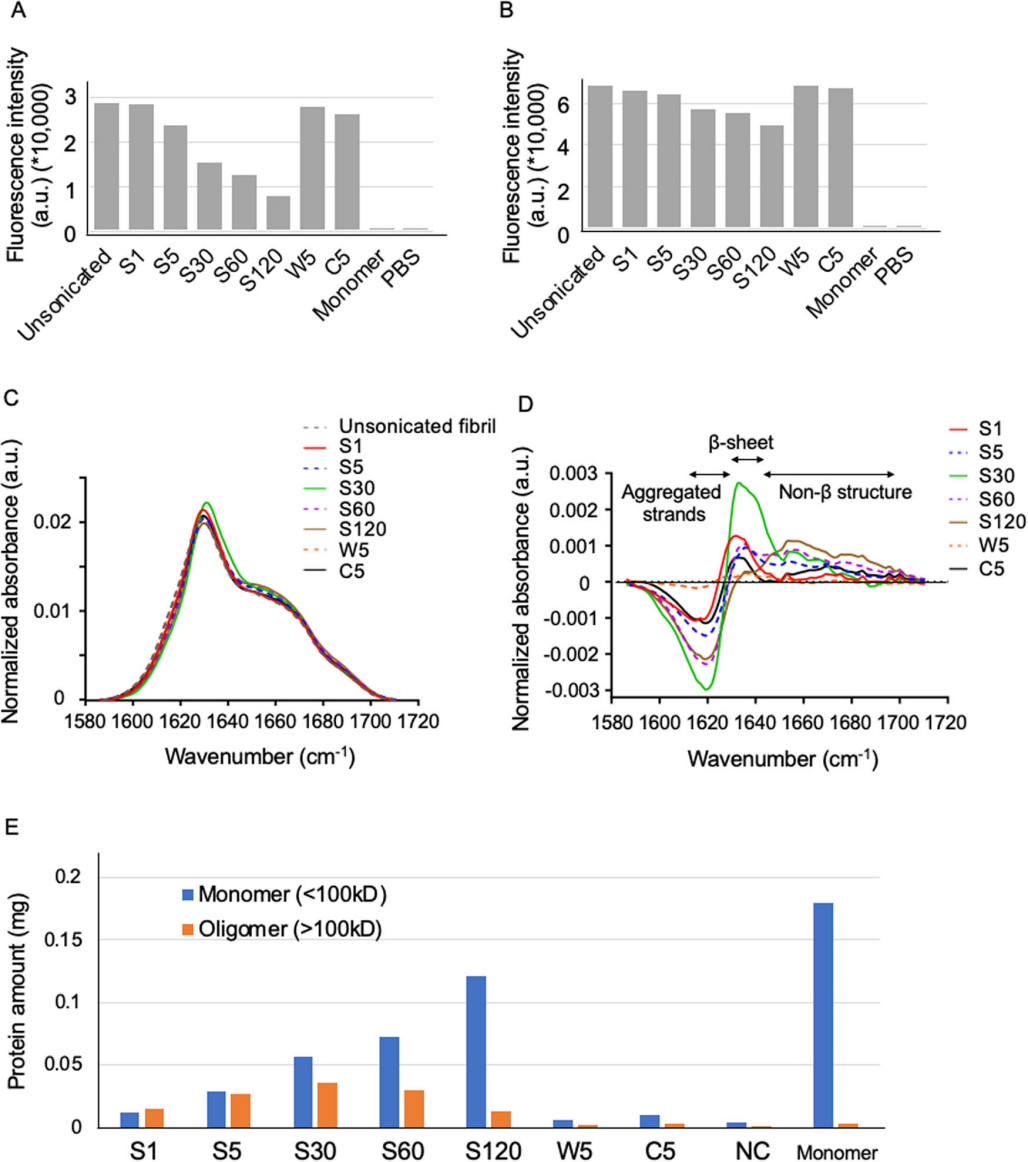
Structural properties of sonicated mPFFs that contribute to differences in seeding activity. **A** A thioflavin T fluorescence assay for unsonicated and sonicated mPFFs, monomer, and PBS. **B** A 4,4’-Dianilino-1,1’-Binaphthyl-5,5’-Disulfonic Acid (Bis-ANS) fluorescence assay for unsonicated and sonicated mPFFs, monomer, and PBS. **C** Area-normalized FTIR-ATR spectra of sonicated and unsonicated mPFFs in the amide I region at 37 °C. FTIR-ATR, Fourier-Transform Infrared-Attenuated Total Reflection. **D** Difference FTIR-ATR spectra (sonicated—unsonicated) calculated from (**C**). Note that sonication for up to 30 min results in a decrease in aggregated strands (around 1620 cm^−1^) and an increase in β-sheets (around 1630 cm^−1^), suggesting that aggregated strands are destroyed and new fibril ends appear, thus explaining the increased seeding activity. **E** Evaluation of the monomer release from mPFFs by sonication. Note that the released monomer fraction (<100kD) increased proportionally to the intensity of sonication, reaching a maximum at 120 min of sonication.

First, ThT and Bis-ANS fluorescence measurements were performed to investigate the β-sheet structure of mPFFs, while ThT fluorescence measurements were conducted for hPFFs^27^. No notable signal changes were observed under the weak condition of the high-power sonicator (W5) or with sonication by the conventional sonicator (C5). However, under the strong condition of the high-power sonicator, the signal decreased in a time-dependent manner (Figs. 5A, B, S4A). Because sonication has been reported to disrupt the amyloid structure^28^, strong sonication may disrupt the β-sheet structure, resulting in reduced seeding activity. However, the difference between S30 and S60 was not as pronounced as the difference between S5 and S30, and thus could not fully explain the decrease in seeding activity due to sonication for 60 min or more.

Next, FTIR spectroscopy was performed on the mPFFs with and without sonication. FTIR spectroscopy uses infrared light to study the vibrational modes of molecules^29,30^. Figure 5C shows the area-normalized FTIR-ATR spectra of the mPFFs in the amide I region. These spectra exhibited similar peaks at approximately 1630 cm^–1^ and shoulders at approximately 1660 cm^–1^, in accordance with a previous report^31^, indicating that the overall secondary structures of mPFFs were maintained during sonication. However, the difference spectra (sonicated—unsonicated) clearly showed subtle changes caused by sonication (Fig. 5D). Sonication for up to 30 min reduced the intensity at approximately 1620 cm^−1^ and simultaneously increased the absorption at approximately 1630 cm^−1^ in a time-dependent manner. This suggests that the strong interchain hydrogen bonds, which are typical of aggregated strands, were converted into weaker hydrogen bonds^32,33^, implying a decrease in aggregated strands (approximately 1620 cm^−1^) and an increase in β-sheets (approximately 1630 cm^−1^). The changes triggered by sonication for up to 30 min can be interpreted as the breakdown of aggregated strands and appearance of new fibril ends, which can explain the enhanced seeding activity. In contrast, longer sonication beyond 30 min resulted in conversion of approximately 10% of β-sheet structures into non-β-sheet structures (0.02 in Fig. 5C vs. 0.002 in Fig. 5D), which exhibited broad absorption in the range of 1640–1700 cm^–1^, leading to decreased seeding activity^34,35^. In C5, both the decrease in aggregated strands (around 1620 cm^-1^) and increase in β-sheets (around 1630 cm^−1^) were insufficient compared to S5 and S30.

Furthermore, considering the possibility that sonication of PFFs releases monomers and affects seeding activity^34^, we investigated the amount of monomers released by sonication^35^. The released monomer fraction (<100kD) of both mPFFs and hPFFs increased proportionally to the intensity of sonication, reaching a maximum at 120 min of sonication (Figs. 5E, S4B), and these monomers lost ThT activity (data not shown). In contrast, the soluble oligomer fraction (>100 kD) increased, reaching a peak at 30 min and then decreasing by 120 min in case of mPFFs (Fig. 5E). The behavior of hPFFs differed slightly from that of mPFFs, with peak values observed at S30 and S60, followed by a slight decrease at S120. However, the reduction at S120 was less pronounced than that seen with mPFFs. C30 showed an increase compared to C5. These monomer release experiments were performed twice to ensure reproducibility.

Finally, to determine whether sonication causes overall structural changes in PFFs, we examined changes in their resistance to proteinase K. The pattern of proteinase K resistance of α-syn mPFFs was examined by treating mPFFs with proteinase K at two different concentrations (0.001 and 0.01 µg/mL) (Fig. S5). The results showed the same band pattern for all mPFFs. Specifically, the signal of the fourth band from the top was the most intense in all mPFFs after 0.001 µg/mL proteinase K treatment, confirming that the folding of α-syn mPFFs was largely unaffected by the intensity of sonication. In this experiment, a decrease in the reactivity of mPFFs that underwent prolonged sonication to the anti-α-syn antibody was observed. To investigate the reason for this, the sonicated mPFFs were subjected to electrophoresis and stained with CBB, revealing a decrease in the molecular weight of mPFFs with prolonged sonication. It was speculated that this intramolecular cleavage might partially contribute to the decrease in seeding activity of PFFs with prolonged sonication.

## Discussion

α-syn PFFs have been frequently employed to generate α-syn propagation models of PD. Sonication is required for the in vivo propagation of PFFs, indicating that their size plays a crucial role in efficient propagation^17,21,36,37^. Furthermore, the duration of sonication has been reported to be directly proportional to the formation of shorter fibrils and increased seeding activity^36^. An examination of sonication time ranging from 0 to 180 s demonstrated that longer sonication time of PFFs resulted in shorter fibrils, higher ThS binding activity, and increased seeding activity, both in vitro and in vivo. However, the effects of sonication beyond 5 min and the possibility of disruption of the β-sheet structure have not yet been investigated. In the present study, we investigated the effect of sonication on the structure of PFFs and their seeding activity. We found that strong sonication with a high-power sonicator resulted in significantly shorter fibrils than sonication with a conventional water bath sonicator. In vitro and in vivo experiments showed that seeding activity increased in a time-dependent manner up to 30 min of sonication under strong conditions, but decreased with prolonged sonication beyond 30 min.

FTIR spectroscopy demonstrated that the linear increase in seeding activity by sonication for up to 30 min could be explained by the breakdown of aggregated strands and generation of new fibril ends. However, prolonged sonication for more than 30 min caused the breakdown of β-sheet structures, resulting in decreased seeding activity. We also observed a time-dependent decrease in the ThT signal of PFFs after strong sonication with a high-power sonicator for more than 5 min. ThT binding to amyloid fibrils depends on a flat β-sheet surface composed of at least five β-strands^38^. A 10% loss in β-sheet structure would significantly reduce ThT binding, as the generation of non-β-sheet structures also perturbs surrounding β-sheet structures and eliminates their flatness. In contrast, seeding activity did not decrease in parallel with the ThT signal and was strongest in S30. Our FTIR analysis revealed that the β-sheet structure exhibited the highest abundance in S30 but decreased in S60, possibly explaining the reduction in seeding activity observed in S60. These findings suggest that sufficient new fibril ends are generated in S30 but are destroyed in S60. The lower seeding activity observed in C5 than in S5 can be attributed to inefficient production of fibril ends, as evidenced by the smaller increase in absorption at approximately 1630 cm^–1^ (Fig. 5D).

The monomer release experiment showed that the released monomer without ThT activity increased proportionally to the intensity of sonication, reaching a maximum at 120 min of sonication, while the soluble oligomer fraction increased, reaching a peak at 30 or 60 min and then decreasing by 120 min (Figs. 5E, S4B). This oligomer fraction appears to correlate with in vitro seeding activity, and may serve as a useful indicator for determining optimal sonication conditions. However, in vivo results demonstrated a marked reduction in propagation under intense sonication conditions (e.g., S60, S120). Unlike in vitro conditions, the in vivo environment has several clearance systems, such as interstitial fluid drainage—including the glymphatic system—as well as phagocytic clearance by microglia and astrocytes. It is reported that particles smaller than 20 nm are more prone to elimination via these drainage pathways^39,40^. Therefore, it is plausible that PFFs fragmented to below 20 nm by intense sonication—some of which may be undetectable by electron microscopy—were efficiently cleared in vivo. This may partially account for the more pronounced reduction in the seeding activity of S60 observed in vivo, and predictions based solely on the biophysical properties of PFFs may be limited in vivo.

**Fig. 6:**
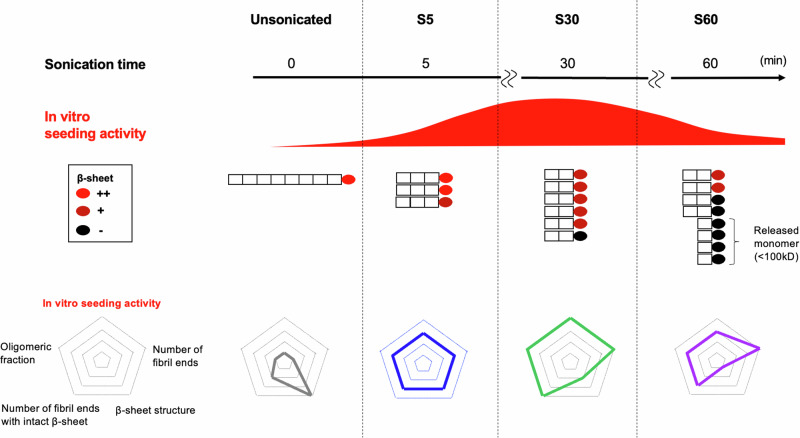
The relationship between the structural properties of sonicated PFFs and their seeding activities. The seeding activity of sonicated α-syn fibrils is determined by the number of fibril ends with the preserved β-sheet structure. A suitable sonication duration up to 30 min increases seeding activity by augmenting the number of fibril ends with abundant intact β-sheet structure. However, prolonged sonication leads to release of monomers with disrupted β-sheet structure from PFFs, resulting in reduced seeding activity. A spider chart was used to compare S5, S30, S60, and unsonicated PFFs across five axes: seeding activity, number of fibril ends, β-sheet structure, number of fibril ends with intact β-sheet, and oligomers fraction.

Combining these results of dye binding assay, FTIR analysis and monomer release experiment suggests that sonication reduces the size of PFFs while exposing the β-sheet structure, thereby increasing seeding activity. However, further sonication increases the amount of released monomer with disrupted β-sheet structure and intramolecular cleavage, which reduces seeding activity, highlighting the importance of an optimal degree of sonication (Fig. 6).

Several laboratories have injected α-syn PFFs into rodent brains to create a propagation model relevant to human PD^10,13,41–43^. Although injections have been performed at various sites, including the striatum, OB, and cortex, we chose to inject them into the OB, which is particularly suitable for measuring the extent of propagation. Three weeks after the injection of mPFFs sonicated for 30 min using a high-power sonicator (referred to as S30), p-α-syn-positive aggregates were observed in the hippocampal CA1 region which is primarily connected to the OB^44^. Previous reports did not observe any pathologies in the hippocampus, even one month after injection^13^, indicating that S30 exhibits higher seeding activity than the PFFs used in the previous report.

Injection of PFFs into the gastrointestinal tract of rodents has also been performed in several laboratories^12,14,25^^45–52^. Two previous reports described spread beyond DMX in the rostro-caudal direction^14,46^, while others have not. In the present study, S5 was injected into the mouse stomach, and after a two-month observation period, propagation was limited to DMX, and no propagation to the LC or other brain areas was observed. Similarly, no propagation to the LC was observed even six months after S30 injection (data not shown). One study showed that propagation beyond the DMX was achieved by mouse PFFs sonicated with a hand sonicator for 30 seconds^14^. The average PFF size was 64.7 nm, and α-syn aggregates disseminated throughout a wide area of the brain within a 3-month period following injection into the stomach and duodenum of 3-month-old wild-type mice. Given that the PFFs were significantly larger with shorter sonication time than in the current study, it is plausible that factors other than sonication and PFF size might have contributed to the widespread propagation. The other study used mPFFs or hPFFs that had also been sonicated with a hand sonicator for 5 min, resulting in an average size of less than 50 nm^46^. Injection of hPFFs into the stomachs of wild-type rats resulted in widespread propagation in the brain within 10 weeks, and the severity of pathology was much greater in older rats than in younger rats. In that study, rats may have been more susceptible to PFFs propagation than mice, especially older rats relative to younger rats.

One limitation of this study is that the propagation efficiency over secondarily connected neurons has not been evaluated in mPFFs sonicated for 0–60 min, and there are no long-term observational data (e.g. 1 year) in mice that received gut injections of mPFFs (S5 and W5).

In conclusion, sonication of PFFs with the high-power sonicator for up to 30 min resulted in the production of shorter PFFs with higher seeding activity, but a decrease in seeding activity was seen with sonication for 60 min or more, due to the destruction of the β-sheet structure. To generate PFFs with high seeding activity, it is recommended to employ a sonication time that is sufficient to expose enough fibril ends while minimizing the disruption of the β-sheet structure. Confirmation and optimization of these PFF parameters can help reduce the inter-laboratory and inter-experimental variability in the α-syn propagation model and thus to improve preclinical animal models of PD.

## Methods

### Preparation of recombinant α-syn monomers and preformed fibrils

Mouse and human α-syn PFFs were generated with slight modifications to previous methods^42^. In brief, mouse and human α-syn were expressed in *Escherichia coli* BL21 (DE3) (BioDynamics Laboratory, Columbus, OH, USA) and subsequently purified through boiling and anion exchange using Q Sepharose Fast Flow (GE Healthcare, Chicago, IL, USA). Endotoxins from *E. coli* were eliminated using the ToxinEraser™ Endotoxin Removal Kit (GenScript, Piscataway, NJ, USA). The endotoxin levels were confirmed to be below the detection limit using the LAL Endotoxin Assay Kit (GenScript) (data not shown). The purified α-syn was then diluted to 5 mg/mL in phosphate-buffered saline (PBS) and incubated at 37 °C with constant agitation at 1000 rpm for 7 days. The mPFFs and hPFFs were used promptly after sonication without freezing or pelleting.

### Sonication of α-syn PFFs

A Bioruptor bath sonicator (UCD-200; COSMO BIO, Tokyo, Japan) was used as a conventional method, and sonication was carried out for 5 min: 30 s of sonication followed by a 30 s interval was repeated five times. In the Bioruptor, the energy is distributed throughout the entire water bath. In contrast, Covaris Focused-ultrasonicator S220 (500217; Covaris, Woburn, MA, USA) is designed so that the ultrasonic energy is focused directly into the solution within the tube, and during sonication, a stirrer inside the tube ensures that the ultrasound is applied uniformly. By combining focused ultrasonic energy with high-speed agitation, the S220 enables consistent and efficient sonication, and direct comparison of sonication intensity based on output energy between these two devices is difficult. The effects of sonication under strong conditions (peak incident power, 100 W; duty factor, 30%; cycles per burst, 1000) for 1, 5, 30, 60, or 120 min and weak conditions (peak incident power, 20 W; duty factor, 5%; cycles per burst, 1000) for 5 min were examined.

### Transmission electron microscopy

α-syn PFFs before and after sonication (7 µL) were placed on a 200-mesh carbon-coated copper grid (Nissin EM, Tokyo, Japan). After standing for 1 min, excess solution was removed using filter paper. The PFFs adsorbed on the grid were negatively stained with 1% (w/v) uranyl acetate solution. Electron micrographs were obtained using a transmission electron microscope (JSM-7900F; JEOL Ltd, Tokyo, Japan) at 80 kV.

### Animals

Two-month-old male mice (C57BL/6 J) were obtained from Shimizu Laboratory Supplies Co., Ltd. (Kyoto, Japan) or CLEA Japan, Inc. (Osaka, Japan). All breeding, housing, and experimental procedures were conducted according to the guidelines for animal care at Kyoto University and approved by the Kyoto University Animal Care and Use Committee.

### Proteinase K digestion of sonicated PFFs

Three micro litter of each sonicated α-syn mPFFs were treated with proteinase K at two different concentrations: 0.001 µg/mL and 0.01 µg/mL. Each sample was incubated with proteinase K at 37 °C for one hour. Following proteinase K treatment, the samples were mixed with sodium dodecyl sulfate-polyacrylamide (SDS) buffer and heated at 95 °C for 5 min. The samples were then subjected to SDS-polyacrylamide gel electrophoresis (SDS-PAGE) to separate the protein fragments based on their molecular weight. The separated proteins were transferred onto a nitrocellulose membrane for Western blot analysis. The membrane was blocked with 4% (w/v) paraformaldehyde (PFA) for 30 min at room temperature (RT). The membrane was then incubated overnight at 4 °C with primary antibodies against phosphorylated α-syn (p-α-syn) (1:2500; 610787; BD Biosciences; Becton, US). After washing with a 5% non-fat milk solution in Tris-buffered saline with Tween 20 (TBST), the membrane was incubated with a secondary antibody (1:5000; 12004158; BIORAD; Hercules, US) for 1 h at RT. The protein bands were visualized using ChemiDoc Touch MP imaging system (BIORAD; Hercules, US).

### Primary culture of mouse hippocampal neurons

Primary hippocampal neurons were prepared from E16 ICR mice as previously described^53^. In brief, the hippocampus was dissected under sterile conditions and triturated in Dulbecco’s modified Eagle’s medium supplemented with fetal bovine serum and penicillin-streptomycin. The cells were centrifuged, resuspended in Neurobasal medium with B27, L-glutamine, and penicillin-streptomycin, and plated on poly-DL-ornithine hydrobromide precoated 24-well plates. Half the medium was replaced every 3-4 days, and the cells were cultured under constant conditions of 37 °C, 5% CO_2_ in a humidified incubator.

### Addition of PFFs to primary neurons

Each PFF sample was first dissolved in Neurobasal medium to a concentration of 10 µg/mL. Two weeks after plating neurons, the PFF-containing medium was then added to the cultures using a half-medium change method to achieve a final concentration of 5 µg/mL. Aggregate formation was assessed by immunocytochemistry 3 days after treatment with mPFFs or 1 week after treatment with hPFFs.

### Immunocytochemistry

Immunocytochemistry was performed as previously described with partial modifications^53^. Primary neurons were washed twice with PBS and then fixed with 4% (w/v) PFA in PBS for 20 min. After two additional washes with PBS, the neurons were incubated with PBS/0.1% Triton X-100 for 10 min and then blocked with 3% (w/v) bovine serum albumin in PBS for 1 h at RT. Neurons were incubated with primary antibodies against phosphorylated α-syn (p-α-syn) (1:3000; ab51253; Abcam; Cambridge, UK) for 2 h at RT. After washing with PBS, neurons were incubated with Alexa Fluor 594-conjugated secondary antibodies (1:1000, A11012; Invitrogen) for 1 h at RT. Finally, the neurons were washed with PBS and cover-slipped, and images were acquired using a BZ-X710 microscope (Keyence, Osaka, Japan) at 20× magnification.

The image acquisition settings were consistent for all samples. The areas of p-α-syn-positive pathology were analyzed using the ImageJ software program (1.53a, National Institutes of Health, USA). The average areas of p-α-syn-positive pathology were measured in four fields of view per sample, and the values were averaged for each condition.

### Western blot analysis of primary neurons

Primary neurons were washed twice with PBS and the washed cells were then lysed directly on the culture dishes. The lysate was collected and centrifuged at 15,000 rpm for 2 min. The pellet was collected and lysed using a lysis buffer containing 10×PBS and 10% SDS, supplemented with protease and phosphatase inhibitors. The samples were sonicated and centrifuged again at 15,000 rpm for 30 min. Equal amounts of protein from each sample were mixed with 0.2 M dithiothreitol, boiled for 5 min, and subjected to SDS-PAGE. Proteins were separated on 10%–20% (w/v) gradient gels (FUJIFILM Wako Pure Chemical Corporation; Osaka, Japan) and transferred onto nitrocellulose membranes. The membranes were treated with 4% (w/v) PFA in PBS for 30 min at RT. After blocking for 1 h with 5% [w/v] skim milk in TBST, the membranes were incubated with primary antibodies against β -actin (1:5000; A5441; Sigma-Aldrich; St. Louis, MO), and p-α-syn (1:5000; ab51253; Abcam; Cambridge, England) overnight at 4 °C. Subsequently, the membranes were incubated with two secondary antibodies (1:5000; 12004162; BIORAD; Hercules, US, and 926-32210; LI-COR; NE, US) for 1 hour at RT.

### Immunohistochemistry

Immunohistochemistry (IHC) was performed as previously described with some modifications^42,^^53^. Mouse brains were perfused with 4% (w/v) PFA in PBS, removed, and then immersed in 4% (w/v) PFA in PBS. Paraffin sections were used for IHC analyses. After being replaced with 70% ethanol, the brains were embedded in paraffin, and 8 μm sections were prepared using an HM 325 rotary microtome (MICROM; PHC Corporation, Tokyo, Japan). The sections were incubated overnight at 4 °C with the anti-phosphorylated α-syn antibody (p-α-syn; 1:5000, ab51253; Abcam), followed by appropriate polymer secondary antibodies (Histofine Simple Stain mouse MAX-PO (R); Nichirei Biosciences, Tokyo, Japan). The sections were washed 3 times for 5 min with PBS between steps, visualized using a peroxidase stain DAB kit (25985-50; Nacalai Tesque, Kyoto, Japan), and examined under a microscope (BX43; OLYMPUS, Tokyo, Japan). The areas of p-α-syn-positive pathology were quantified in specific brain regions, including the anterior olfactory nucleus (AON), piriform cortex, hippocampus (CA1), areas around the nasal cavity, and dorsal motor nucleus of the vagus nerve (DMX) in coronal sections, using the ImageJ software program.

### Evaluation of monomer release by sonication

This experiment is based on the previous report with slight modification^35^. Each sonicated α-syn PFF sample was centrifuged at 100,000 × *g* for 30 min at 24 °C to separate the fibrillar species from the soluble species. The supernatant, containing the soluble α-syn species (including monomer fractions), was carefully collected and subjected to filtration using a 100 kD molecular weight cut-off membrane (Amicon Ultra; UFC510096; Merck, MA, US). The filtration process was conducted at 14,000 × *g* for 10 min. The filtrate primarily contained the monomer fractions, while the retentate contained the soluble oligomer fractions. The protein content of each fraction was measured using the BCA assay.

### Thioflavin T and Bis-ANS fluorescence assay

Thioflavin T (ThT) and Bis-ANS were added to the α-syn PFF solution at a final concentration of 5 μM. After incubation at RT for 30 min, the fluorescence intensity was measured using a multi-mode plate reader (ARVO X3; PerkinElmer, Shelton, USA). The excitation wavelengths for the ThT and Bis-ANS assays were 450 nm and 355 nm, respectively, and the emission wavelength was 535 nm for both assays.

### Fourier transform infrared-attenuated total reflection (FTIR-ATR) spectra

The samples were applied to a germanium ATR plate (70 mm × 10 mm × 5 mm) under exposure to a stream of nitrogen gas. To eliminate residual water, the samples were subjected to a vacuum overnight using P_2_O_5_. FTIR-ATR measurements were conducted at 37 °C using a Bruker Optics TENSOR 27 spectrometer (Bruker Corporation, Billerica, MA, USA). The total number of reflections on the sample side was nine. The spectra were acquired with a resolution of 2 cm^–1^ and an angle of incidence of 45°, derived from 256 coadded interferograms. Before the frequency measurement, gently sloping water vapor was subtracted to enhance the background. ATR correction was performed using refractive indices of 4.003 and 1.7 for germanium and protein, respectively.

### Stereotactic surgery

Stereotaxic injection was conducted with minor modifications to previous methods^50^. Mice anesthetized with sevoflurane were stereotaxically injected with 0.5 μL of α-syn PFFs (5 mg/mL) into the left olfactory bulb (OB) using a 33-gauge Neuros syringe (Hamilton, Reno, NV).

### Intranasal administration of α-syn PFFs

α-syn mPFFs were intranasally administered to sevoflurane-anesthetized mice as described previously^16^. Mice were positioned in the supine posture, and mPFFs (5 mg/mL) were instilled into the left nasal cavity. Subsequently, 1 μL of mPFFs was administered at 1 min intervals to ensure adequate absorption into the mucosa. In total, 25 μL of mPFFs was administered into the left nasal cavity.

### Gut injection of α-syn PFFs

α-syn mPFFs were injected into the stomachs of mice with minor modifications to previous methodologies^42^. A 2 cm incision was created along the abdominal midline, followed by the administration of mPFFs into the gastric wall. Each of 5 sites of gastric wall (3 on the fenestra side and 2 on the pylorus side) was injected with 2.5 µL of α-syn PFFs (5 mg/mL). using a 37-gauge needle (Saito Medical Instruments, Tochigi, Japan) attached to 10 µL syringe (Hamilton, Reno, NV).

### Statistical analyses

Statistical analyses were performed using the PRISM statistical software package (SAS Institute, NC, USA). Statistical significance was assessed using a one-way analysis of variance with Tukey’s multiple comparisons test. The Mann-Whitney U test was used to compare the data between the two groups. Differences with *p*-values less than 0.05 were considered statistically significant.

## Supplementary information

Supplementary information

## Data Availability

The datasets utilized and examined during the present study are available from the corresponding author upon reasonable request.
