# Supplementary material for: Impact of α-synuclein fibril structure on seeding activity in experimental models of Parkinson’s disease
Source: NPJ Parkinsons Dis. 2025 Jul 31;11:224. doi: 10.1038/s41531-025-01080-2 (PMC12313852; doi:10.1038/s41531-025-01080-2)
Supplement: Supplementary file 1 — Supplementary information [file 41531_2025_1080_MOESM1_ESM.pdf]

Supplementary figure 1 Transmission electron microscopy of sonicated α-syn hPFFs reveals that the high-power sonicator produces much shorter PFFs than the conventional sonicator, similar to the findings observed with mPFFs

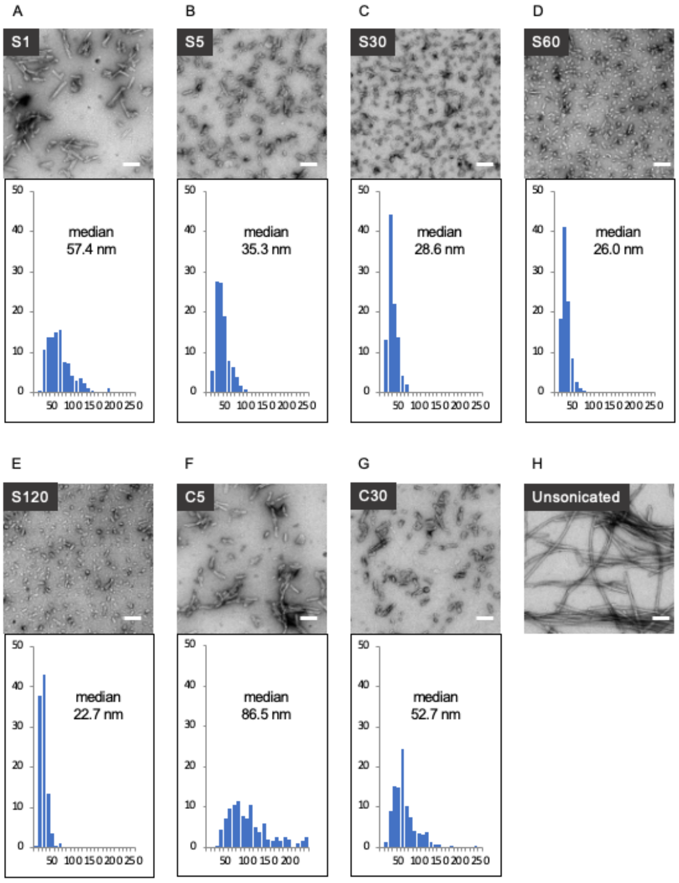

(A–E) Human α-syn PFFs sonicated for 1 minute (S1), 5 minutes (S5), 30 minutes (S30), 60 minutes (S60), or 120 minutes (S120) under strong conditions using a high-power sonicator. (F, G) PFFs sonicated for 5 minutes (C5) and 30 minutes (C30) using a conventional bath sonicator. (H) Unsonicated PFFs. (A–G) Histograms and median PFF lengths are shown below the corresponding electron microscope images. Scale bars: 100 nm.

Supplementary figure 2 Shorter α-syn hPFFs show a higher seeding activity in primary neurons.

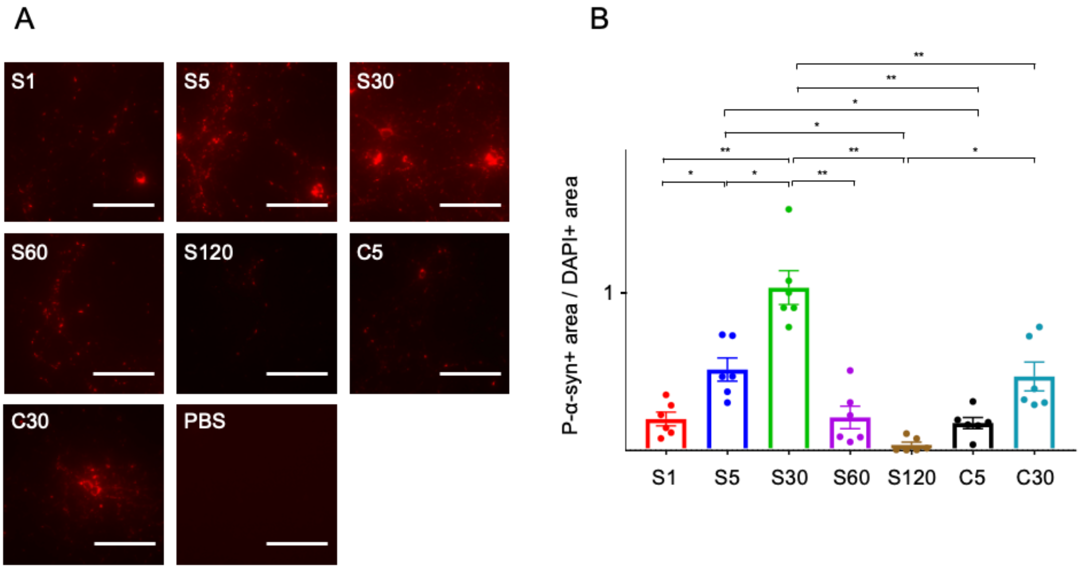

(A) Representative images of α-syn aggregates in mouse primary hippocampal neurons one week after the addition of each hPFF, using an antibody against p-α-syn. Scale bars: 100 nm. (B) P-α-syn positive area divided by the DAPI-positive area to normalize for the number of cells. Each plotted dot represents the average from four independent well regions, and six independent experiments were analyzed. A one-way ANOVA with Tukey's multiple-comparisons test is performed; *p <0.05, **p <0.0001. Data are expressed as the mean ± SEM.

Supplementary figure 3 Full western blot of Fig. 2C

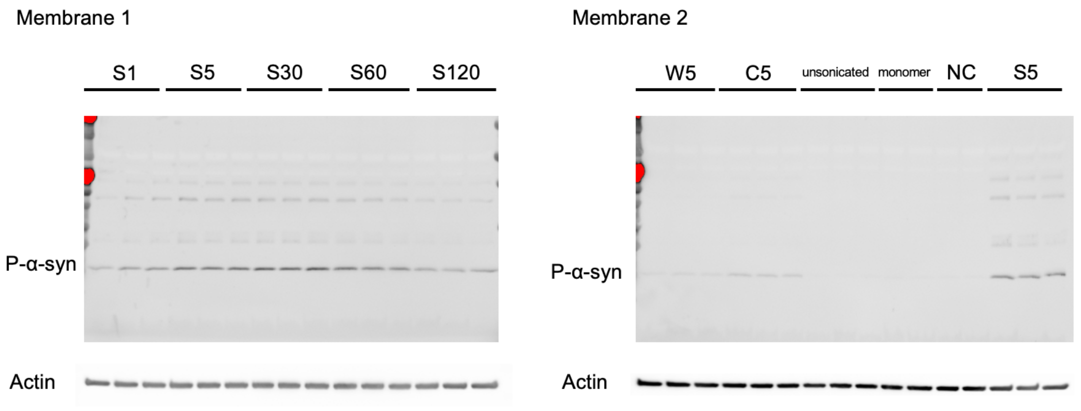

Supplementary figure 4 Structural properties of sonicated hPFFs

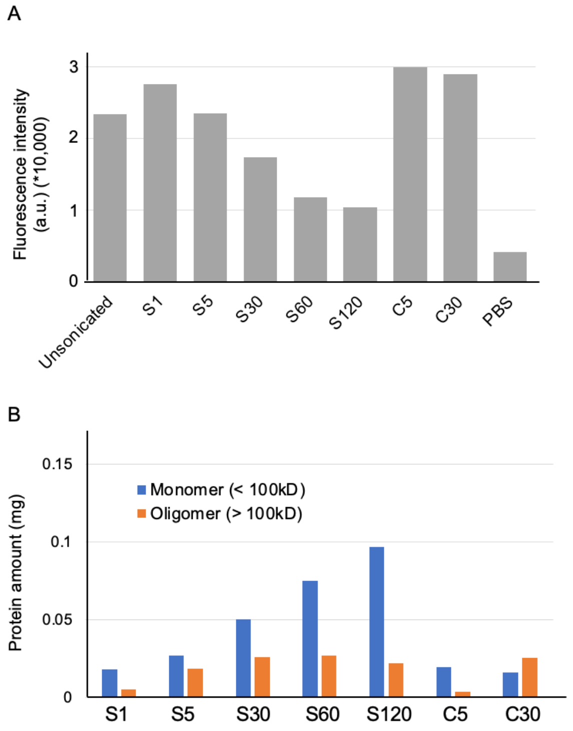

(A) A thioflavin T fluorescence assay for unsonicated and sonicated hPFFs and PBS. (B) Evaluation of the monomer release from hPFFs by sonication.

Supplementary figure 5 Proteinase K digestion and CBB staining of PFFs with varying sonication times

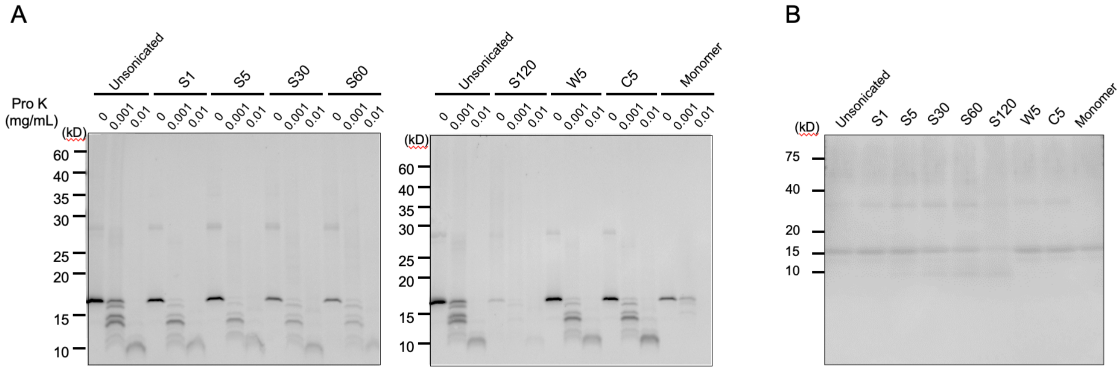

(A) The Proteinase K digestion pattern of α-syn mPFFs is evaluated by exposing them to two concentrations of Proteinase K (0.001 and 0.01 μg/mL) and comparing them with unsonicated mPFFs. The band patterns are consistent across all sonicated mPFFs. (B) Sonicated α-syn mPFFs were subjected to electrophoresis and stained with CBB. Note the α-syn species with a reduced molecular weight, especially in the mPFFs subjected to prolonged sonication.
